# Effect of the MgO/Silica Fume Ratio on the Reaction Process of the MgO–SiO_2_–H_2_O System

**DOI:** 10.3390/ma12010080

**Published:** 2018-12-26

**Authors:** Zhaoheng Li, Yudong Xu, Hao Liu, Jianwei Zhang, Jiangxiong Wei, Qijun Yu

**Affiliations:** 1 School of Materials Science and Engineering, South China University of Technology, Guangzhou 510640, China; lizhaoheng2008@163.com (Z.L.); xyd19881105@126.com (Y.X.); mshliu@mail.scut.edu.cn (H.L.); concyuq@scut.edu.cn (Q.Y.); 2 Guangdong Research Institute of Water Resources and Hydropower, Guangzhou 510635, China; 3 School of Water Conservancy, North China University of Water Resources and Electric Power, Zhengzhou 450046, China; zjwcivil@126.com

**Keywords:** MgO, silica fume, reaction process, thermodynamics, kinetics

## Abstract

In order to clarify the effect of the MgO–silica fume (SF) ratio on the reaction process of the MgO–SiO_2_–H_2_O system, the reaction products and degree of reaction were characterized. Furthermore, the parameters of the reaction thermodynamics were calculated and the reaction kinetics were deduced. The results indicate that a large amount of Mg(OH)_2_ and small quantities of magnesium silicate hydrate (M–S–H) gels were generated upon dissolution of MgO. However, the M–S–H gels were continuously generated until the SF or Mg(OH)_2_ was consumed completely. For a MgO dosage less than 50% of the total MgO–SiO_2_–H_2_O system, the main product was M–S–H gel, while for a MgO dosage greater than 50%, the main product was Mg(OH)_2_. The results indicate that M–S–H gels have greater stability than Mg(OH)_2_, and the final reaction product was prone to be M–S–H gels. Based on the experimental values, an equation is proposed for the reaction kinetics of MgO.

## 1. Introduction

The MgO–SiO_2_–H_2_O system was developed as a novel cementitious material [1,2,3,4,5]. Numerous applications have been realized, including usage in thermal insulation material [6], waste sealing material [7], refractory material [8], and soil stabilization [9]. Magnesium silicate hydrate (M–S–H) gel is the typical reaction product formed in the MgO–SiO_2_–H_2_O system [7,8,9,10,11,12,13,14]. Cole [15] found a crystalline M–S–H phase, which was identified as 4MgO·SiO_2_·8.5H_2_O. Gollop [16] found that M–S–H gel forms in Portland cement pastes by magnesium sulfate attack, and characterized the gel as a poorly crystallized serpentine (3MgO·2SiO_2_·2H_2_O). Brew [17,18] characterized chemically synthesized M–S–H gels and investigated the incorporation of cesium and potassium. Vandeperre et al. [19] discovered that brucite (Mg(OH)_2_) could react with the amorphous silica present in pulverized fuel ash to form M–S–H gels. In fact, the reaction products containing Mg were found to be Mg(OH)_2_ and hydrotalcite-like phases, rather than M–S–H gels [20]. In addition, because magnesium is frequently associated with calcium in carbonate rocks (the raw material for cement production) [21], the existence of MgO in cement-based materials is inevitable. Overall, the reaction process of MgO in the MgO–SiO_2_–H_2_O system and the effects of the MgO/SF ratio on the reaction process are not clearly understood, and further research is necessary to clarify these reactions.

In this study, the reaction processes and reaction products of MgO/silica fume (SF) pastes were characterized. Further, thermodynamic calculations and kinetic deductions were conducted, and the effects of the MgO/SF ratio on the reaction process of the MgO–SiO_2_–H_2_O system clarified. The results contribute to a better understanding of the reaction processes of the MgO–SiO_2_–H_2_O system and the development of a novel cementitious material.

## 2. Materials and Methods

### 2.1. Raw Materials

**Figure 1:**
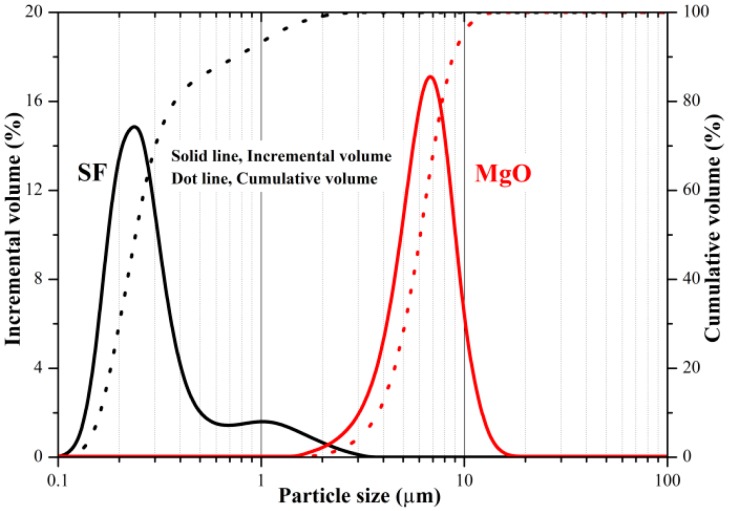
Particle size distributions of MgO and SF powders. Reprinted with permission from [3]; Copyright 2014 Elsevier.

The chemical compositions of MgO and SF [3], as determined by X-ray fluorescence spectroscopy (XRF, PANalytical, Almelo, The Netherlands), are listed in Table 1. The particle size distributions of MgO and SF powders [3], as measured by laser diffraction (Partica LA-950V2, HORIBA, Kyoto, Japan) are presented in Figure 1.

### 2.2. Sample Preparation

#### 2.2.1. Preparation of MgO/SF Pastes

According to the proportions of the mixture listed in Table 2, MgO/SF pastes were prepared by mixing reactive MgO, SF, and water homogeneously, followed by sealing of the mixture in polyethylene bags and curing at room temperature (25 ± 1 °C) [3].

#### 2.2.2. Preparation of M–S–H Gel

M–S–H gel with a Mg/Si ratio of 1.0 was synthesized as reported in [3]. In accordance with [17], Mg(NO_3_)_2_·6H_2_O solution was slowly added to a Na_2_SiO_3_·5H_2_O solution by stirring in a flask at 0 °C. The precipitated composite was filtered and washed using ultrapure water [3].

### 2.3. Testing Methods

#### 2.3.1. Measurement of Mechanical Strength

The mechanical strength of MgO/SF mortar specimens (40 × 40 × 160 mm^3^) were measured according to ASTM C 349 at each curing age (days 3, 7, 28, and 90) [22].

#### 2.3.2. Measurement of Heat Evolution

The heat evolution of the MgO/SF pastes [3] was measured up to day 10 at 25 °C using a TAM-Air isothermal calorimeter (Thermometric AB, Jarfalla, Sweden) according to ASTM C 1702-09 [23].

#### 2.3.3. Characterization of the Reaction Products

MgO/SF pastes cured at day 1, 3, 7, 28, and 90 were frozen (−195 °C) by immersion in liquid nitrogen and then dried via the freeze-drying technique [24]. The reaction products were characterized by using X-ray diffraction (X’Pert Pro diffractometer, Cu Kα_1_, 40 kV and 40 mA; Philips, Amsterdam, The Netherlands) and thermal analysis (Netzsch STA 449C, 10 °C/min in N_2_ atmosphere; Netzsch, Bavaria, Germany) [3].

#### 2.3.4. Measurement of MgO Content

The residual MgO content in MgO/SF pastes was determined by quantitative X-ray diffraction (Q-XRD, Cu Kα_1_, 40 kV and 40 mA; PANalytical B.V., Almelo, The Netherlands) using the *K*-value method [25], in which ZnO was used as the internal standard material.

#### 2.3.5. Calculation of Mg(OH)_2_ and M–S–H Gel Contents

The contents of Mg(OH)_2_ and M–S–H gel were calculated according to the weight losses displayed in the DTG/TG curves, as shown in Equations (1)–(3) [3,4].

(1) C[Mg(OH)2]=ΔM3×M[Mg(OH)2]M[H2O]

(2) $$C{\left[\mathrm{PAW}\right]}_{M-S-H\;\mathrm{gel}}=\Delta M_{1}$$

(3) $$C{\left[\mathrm{CBW}\right]}_{M-S-H\;\mathrm{gel}}=\Delta M_{2}-\Delta M_{3}-\Delta M_{4}$$

where *C*_[Mg(OH)₂]_ is the Mg(OH)_2_ content; *C*[PAW]_M–S–H gel_ and *C*[CBW]_M–S–H gel_ are the contents of the M–S–H gel (wt.%), characterized by the physically absorbed water (PAW) and chemically bound water (CBW), respectively; *M*_[Mg(OH)₂]_ and *M*_[H₂O]_ are the molar masses of Mg(OH)_2_ (g/mol) and H_2_O (g/mol), respectively; Δ*M*_1_ and Δ*M*_2_ are the weight losses in the range of 50–200 °C and 200–1000 °C in TG curves, respectively; Δ*M*_3_ and Δ*M*_4_ are the weight losses due to the dehydration of Mg(OH)_2_ and decomposition of MgCO_3_, as shown in Section “Mg(OH)_2_ Content”.

#### 2.3.6. Degree of Reaction

The degree of reaction (α) of MgO was calculated using Equation (4).

(4) α=C0(MgO)−Ct(MgO)C0(MgO)

where *C*_0_(MgO) and *C_t_*(MgO) are the mass ratios of MgO in MgO/SF pastes cured at 0 and *t* days, respectively, which are normalized using the residual mass ratio of the samples following calcining at 1000 °C.

## 3. Results and Discussion

### 3.1. Reaction Process

#### 3.1.1. Compressive Strength and Flexural Strength

**Figure 2:**
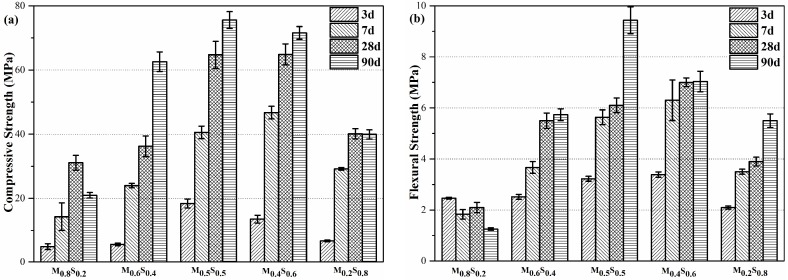
Mechanical strength of MgO/SF mortar: (**a**) compressive strength; (**b**) flexural strength.

Figure 2 shows the compressive strength and flexural strength development of MgO/SF mortar up to 90 days. The compressive strength and flexural strength increased with curing age. However, because MgO hydrated too slowly, it was not able to gain sufficient strength after day 28 and strength retrogression occurred even for M_0.8_–S_0.2_ 90 days later. The main reason for this is that MgO produces a large amount of Mg(OH)_2_ in the later stage of the reaction. The gelling ability of Mg(OH)_2_ is poor and volume expansion occurs simultaneously, which leads to a decrease in strength. The incorporation of SF with MgO resulted in higher strength because of the reaction between them, and strength increased with the increase in SF amount. M_0.5_–S_0.5_ mortar has the highest strength; its compressive strength at days 3, 7, 28, and 90 is 18.4, 40.6, 64.7 and 75.6 MPa, and its flexural strength is 3.2, 5.6, 6.1 and 9.4 MPa, respectively. This phenomenon can be attributed to the fact that when the MgO content is low, the amount of M–S–H gel generated is small and the strength is low. When the MgO content is high, the reaction products are mainly Mg(OH)_2_, and the mechanical properties are poor. In general, the strength increased with curing time up to 90 days. However, it was found that further curing did not lead to any increase in strength; on the contrary, the strength sometimes decreased.

#### 3.1.2. Heat Evolution

**Figure 3:**
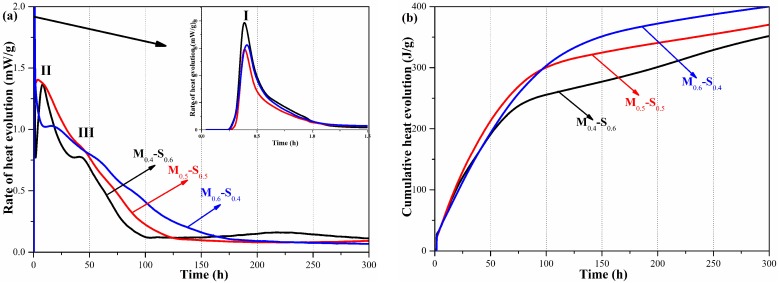
Heat evolution of MgO/SF pastes: (**a**) rate of heat evolution; (**b**) cumulative heat.

Three peaks (designated as I, II, and III, at approximately 0.5, 12, and 48 h) [5] were observed in the rate of heat evolution curves of the MgO/SF pastes (Figure 3a) [3]: the initial peak (I) is related to the exothermic wetting of the mixtures [5]; the second peak (II) corresponds to the formation of Mg(OH)_2_; the third peak (III) is attributed to the formation of the M–S–H gel [5]. As the MgO content decreased, the second peak became less distinct, while the third peak broadened (Figure 3a), resulting in a decrease in the early cumulative heat. The cumulative heat of the M_0.4_S_0.6_ paste (255.88 J/g) was lower than that of the M_0.5_S_0.5_ paste (299.06 J/g) and M_0.6_S_0.4_ paste (302.94 J/g) at an early curing age (100 h), while at an advanced curing age (300 h), the cumulative heats were 351.83, 370.32 and 400.05 J/g, respectively. The results indicate that the formation of Mg(OH)_2_ is restrained by increasing the content of SF, which also confirms that the formation of the M–S–H gel is a continuous process [5].

#### 3.1.3. Variation in the Composition of MGO/SF Pastes

**Figure 4:**
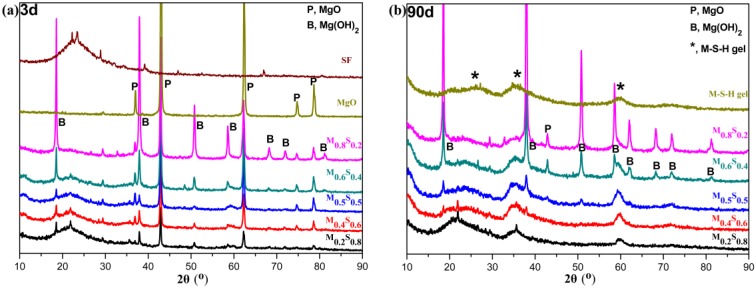
XRD patterns of MgO/SF pastes, MgO, SF and synthetic M–S–H gel: (**a**) M–S–H gel and SF, cured for 3 days; (**b**) M–S–H gel and SF, cured for 90 days.

The XRD patterns of synthetic M–S–H gel and SF (Figure 4) contain broad peaks in the range of 23°–28°, 32°–38°, and 58°–62° [6], all of which correspond to the M–S–H gel. Another broad peak exists at 15°–25° and is attributed to amorphous SiO_2_ [3,26]. In addition, the sharp peaks at 36.9°, 42.9°, 62.3°, 74.7°, and 78.6° are attributed to MgO [5] and the peaks at 18.6°, 38.0°, 50.8°, and 58.6° correspond to Mg(OH)_2_ [5]. The Mg(OH)_2_ peaks appeared in the XRD patterns of all MgO/SF pastes at an early age (day 3). However, no Mg(OH)_2_ was found in the M_0.2_S_0.8_ and M_0.4_S_0.6_ pastes after day 90 (Figure 4b), and the formation of the M–S–H gel was confirmed. Notably, all MgO fully reacted in the M_0.2_S_0.8_ paste after day 90.

#### MgO Content

**Figure 5:**
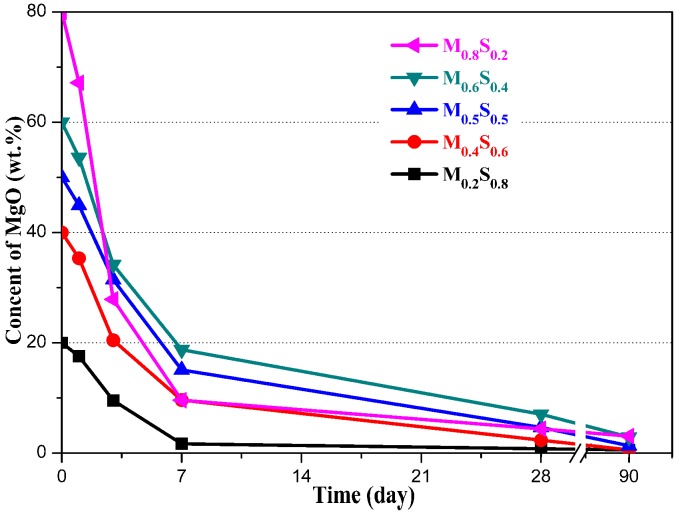
The MgO content in the MgO/SF pastes.

The MgO content was determined via the Q-XRD method; the results are shown in Figure 5. The MgO content rapidly decreased within the first 7 days, after which the rate of decrease lowered. After 90 days, no MgO remained in the M_0.2_S_0.8_ or M_0.4_S_0.6_ pastes, while the content of MgO in the M_0.5_S_0.5_, M_0.6_S_0.4_, and M_0.8_S_0.2_ pastes was 1.34, 2.91, and 3.07%, respectively.

#### Mg(OH)_2_ Content

**Figure 6:**
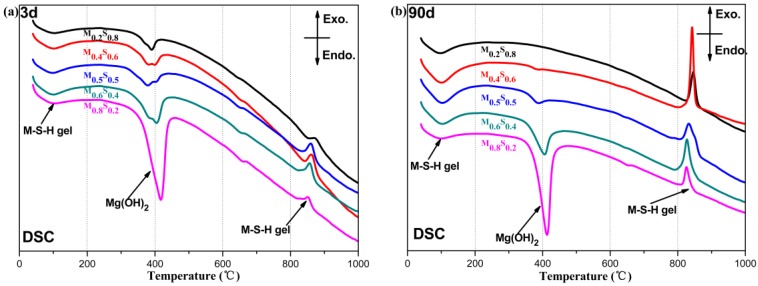
DSC curves of MgO/SF pastes: (**a**) cured for 3 days; (**b**) cured for 90 days.

**Figure 7:**
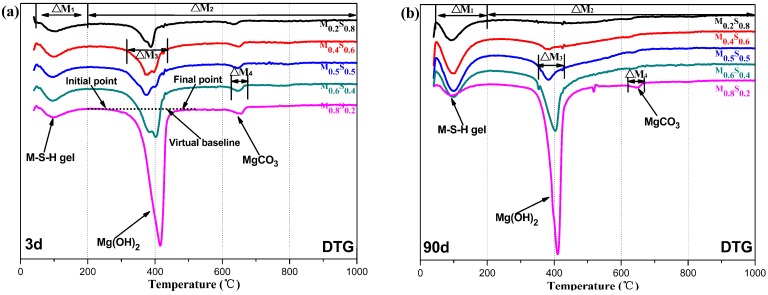
DTG curves of MgO/SF pastes: (**a**) cured for 3 days; (**b**) cured for 90 days.

Figure 6 and Figure 7 show the DSC/DTG curves of the MgO/SF pastes. The first endothermic valley (Δ*M*_1_, ranging from 50–200 °C) corresponds to the weight loss of the PAW of reaction products (mainly products of the M–S–H gel) [3,8]; the second endothermic valley (Δ*M*_3_, at approximately 400 °C) occurs due to the removal of the hydroxyl from Mg(OH)_2_ [4,5,6,7,8]; and, the exothermic effect at approximately 850 °C is associated with the recrystallization of the M–S–H gel [3,12,13,14,15,16]. In addition, Δ*M*_2_ (which is in the range 200–1000 °C) is the weight loss of the CBW from the reaction products (including Mg(OH)_2_, MgCO_3_ and M–S–H gel) [3]. Here, Δ*M*_4_ is the weight loss due to the decomposition of MgCO_3_ [3,5], and the initial and final points are determined by the virtual baseline of the DSC curves (as shown in Figure 7a).

**Figure 8:**
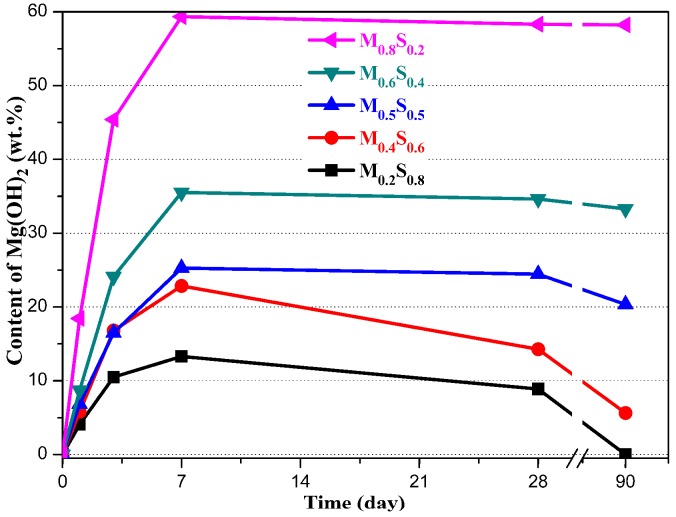
The Mg(OH)_2_ content found in the MgO/SF pastes.

The Mg(OH)_2_ content was calculated using Equation (1), the results are shown in Figure 8. The Mg(OH)_2_ content in the M_0.2_S_0.8_, M_0.4_S_0.6_, M_0.5_S_0.5_, M_0.6_S_0.4_, and M_0.8_S_0.2_ pastes increased to 13.29%, 22.85%, 25.27%, 35.52%, and 59.34% in the first 14 days, respectively, and then slowly decreased. No Mg(OH)_2_ was found in the M_0.2_S_0.8_ paste after 90 days, while a small amount of Mg(OH)_2_ (approximately 5%) was found in the M_0.4_S_0.6_ paste.

#### PAW and CBW Contents

**Figure 9:**
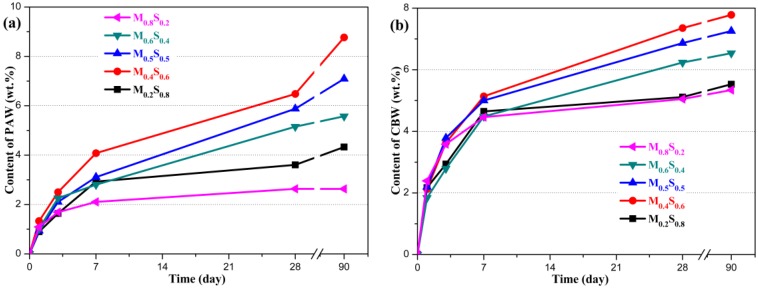
PAW and CBW contents in MgO/SF pastes: (**a**) PAW content; (**b**) CBW content.

Owing to the formation of M–S–H gel, the first endothermic valley gradually became prominent (Figure 6). Therefore, the amounts of PAW and CBW associated with the M–S–H gel (shown in Figure 9) are considered to be indicative of the M–S–H gel content. The amounts of both PAW and CBW continuously increased, which is related to the gradual formation of the M–S–H gel. The amounts of PAW and CBW in the M_0.4_S_0.6_ paste were higher than those in other MgO/SF pastes, which indicated that the amount of M–S–H gel in the M_0.4_S_0.6_ paste is higher than that in the other MgO/SF pastes.

### 3.2. Reaction Thermodynamics

The laws of thermodynamics can predict the probability of a reaction, as well as the final state achieved when the reaction is completed. These laws are often used in analyzing cement-based materials [27,28,29]. Consequently, they were employed in the analysis of the reaction of the MgO–SiO_2_–H_2_O system in this study. The thermodynamic coefficients for the minerals (or species) of the MgO–SiO_2_–H_2_O system are shown in Table 3.

The thermodynamic calculations (standard molar entropy Δ_r_*S*^θ^, standard molar enthalpy of formation ∆_r_*H*^θ^, and standard molar Gibbs free energy Δ_r_*G*^θ^) for the potential reactions of the MgO–SiO_2_–H_2_O system were computed using Equations (5)–(7):

(5) $$\Delta_{r}S^{\theta }=\underset{i}{\sum }v_{i}S_{m}^{\theta }$$

(6) $$\Delta_{r}H^{\theta }=\underset{i}{\sum }v_{i}\Delta_{f}H_{m}^{\theta }$$

(7) $$\Delta_{r}G^{\theta }=\underset{i}{\sum }v_{i}\Delta_{f}G_{m}^{\theta }=-RT\ln K$$

where, ν*_i_* is the stoichiometric reaction coefficient, *R* = 8.314 J/(mol·K), and *T* is the temperature in *K* [3]. Here, ∆_r_*S*^θ^ > 0 indicates that the reaction tends to be more uniform, and ∆_r_*H*^θ^ < 0 indicates that it is an exothermic reaction (reactions occur spontaneously). A chemical reaction occurs spontaneously when ∆_r_*G*^θ^ < 0, and under an isothermal constant pressure condition [3]. Note that a higher *K* value signifies that the degree of reactant conversion is higher and the tendency for a positive reaction to occur is greater [3].

Some of the potential reactions of the MgO–SiO_2_–H_2_O system are listed in Table 4. Based on the ∆_f_*G*_m_^θ^ and ∆_r_*G*^θ^ values, M–S–H phases are more stable than Mg(OH)_2_. Furthermore, the formation of the M–S–H phases is much easier than Mg(OH)_2_, as the ∆_r_*G*^θ^ values of reactions ④ (−63.99 kJ·mol^−1^) and ⑤ (−261.32 kJ·mol^−^^1^) are much higher than reaction ⑥ (−299.58 kJ·mol^−^^1^).

### 3.3. Reaction Kinetics

#### 3.3.1. Theoretical Deduction of the Reaction Kinetics Equation

The reaction process for the MgO−SiO_2_−H_2_O system was divided into two stages, as in references [3,14]. First, MgO reacted with water to form Mg(OH)_2_ and then reacted with dissolved silica to form M−S−H gel [5]. Wei et al. [2,13] calculated the thermodynamics of the MgO−SiO_2_−H_2_O system and confirmed that the formation of M−S−H gel and Mg(OH)_2_ occurred simultaneously. The dissolution of MgO is represented by Equation (8):

(8) $$\mathrm{MgO}+H_{2}O\to {\mathrm{Mg}}^{2+}+2{\mathrm{OH}}^{-}$$

The amount of MgO rapidly decreased at an early age, when the content of MgO was high, whereas the content of MgO decreased slowly when the amount of MgO was low. The reaction rate of MgO in the MgO−SiO_2_−H_2_O system is proportional to the first power of the MgO content, as shown in Equation (9):

(9) $$-\frac{d\left[C_{t}\left(\mathrm{MgO}\right)\right]}{dt}=k\times C_{t}\left(\mathrm{MgO}\right)$$

where *k* is the reaction rate constant.

By integrating both sides, Equation (9) can be modified to Equation (10):

(10) $$\int_{C_{0}\left(\mathrm{MgO}\right)}^{C_{t}\left(\mathrm{MgO}\right)}\frac{d\left[C_{t}\left(\mathrm{MgO}\right)\right]}{C_{t}\left(\mathrm{MgO}\right)}=k\int_{0}^{t}dt$$

Equation (10) can also be written as follows:

(11) $$C_{t}\left(\mathrm{MgO}\right)=C_{0}\left(\mathrm{MgO}\right)\times e^{-kt}$$

The relationship between α and t is shown in Equation (12) (formulated by combining Equations (7) and (11)):

(12) $$\alpha =1-e^{-kt}$$

#### 3.3.2. Verification of the Reaction Kinetics Equation

**Figure 10:**
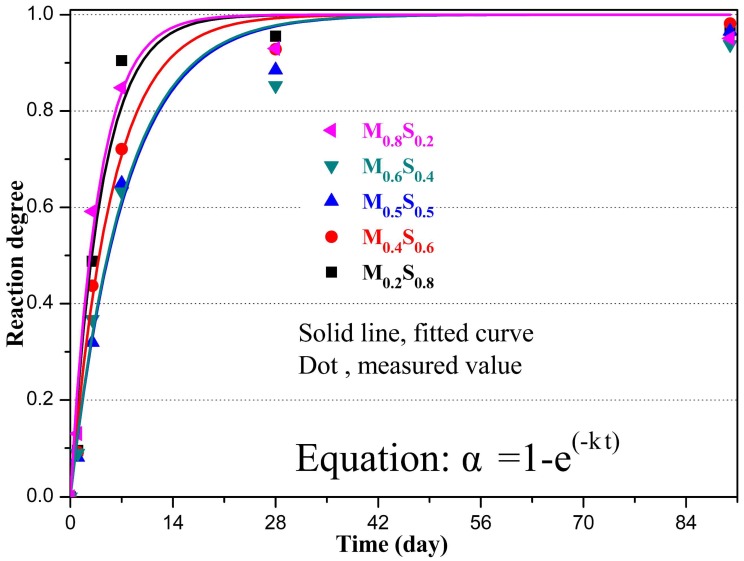
Fitted curves for the reaction kinetics.

The estimated values of α for varying MgO/SF pastes were fitted using the above reaction kinetics equation (Equation (12)). The fitted curves and their parameters are shown in Figure 10 and Table 5, respectively. The results demonstrate that the reaction kinetics follow the well-defined kinetics equation and the correlation is obvious (*R*^2^ > 0.97), which confirms the theoretical deduction process.

The reaction kinetics for MgO in different MgO/SF pastes were calculated using the relation between the degree of reaction (α) and curing age (*t*). The rate constant decreased with increasing MgO content when the dosage of MgO was lower than 50%, and increased with increasing MgO content when the dosage of MgO was higher than 50%.

## 4. Conclusions

When mixed with SF and water, MgO hydrated to form brucite, which immediately reacted with dissolved silica to produce M–S–H gel. The formation of amorphous M–S–H gel at room temperature was confirmed via XRD, TG, and microstructure analyses and the effect of the MgO/SF ratio on the reaction process of the MgO–SiO_2_–H_2_O system was discussed. XRD revealed the components of the reaction products, and TG was found to be a useful tool for determining the quantity of the reaction products. The following conclusions can be drawn from the present study:

- Mg(OH)_2_ results from the dissolution of MgO. M–S–H gels generates from the reaction between Mg^2+^ and hydrated silica, and consequently the dissolution of Mg(OH)_2_ and SiO_2_ is promoted.
- The formation reaction for the M–S–H gel is the main reaction in the MgO–SiO_2_–H_2_O system when the dosage of MgO is lower than 50%, while the formation reaction of Mg(OH)_2_ is the main reaction when the dosage of MgO is higher than 50%.
- Based on the thermodynamic calculations, M–S–H gels are more stable than Mg(OH)_2_. Furthermore, the formation reactions for the M–S–H gels occurred more completely than those for Mg(OH)_2_.
- The reaction kinetics of MgO in the MgO–SiO_2_–H_2_O system conforms to α = 1 − *e*^−*kt*^ (*R*^2^ > 0.97). Because of the decrease in the SF dosage, the rate constant decreased with decreasing SF content when the dosage of MgO was lower than 50%. As a result of the formation rate of the M–S–H gels being lower than Mg(OH)_2_, the rate constant increased with increasing MgO content when the dosage of MgO was higher than 50%.

## Figures and Tables

**Table 1:** Chemical composition of the MgO and SF [3].

Material	Composition (wt.%)								Specific Density (g/cm^3^)	BET Specific Surface Area (m^2^/g)
	SiO_2_	MgO	CaO	K_2_O	Na_2_O	Al_2_O_3_	P_2_O_5_	SO_3_		
MgO	0.29	97.22	1.45	–	–	0.21	0.06	0.20	2.96	51.60
SF	95.74	0.63	1.25	1.07	0.33	0.50	0.20	0.15	2.03	16.04

Note: –, undetected.

**Table 2:** Mixture proportions of MgO/SF pastes.

Sample ID	MgO (wt.%)	SF (wt.%)	W/B Ratio
M_0.2_S_0.8_	20	80	1.0
M_0.4_S_0.6_	40	60	1.0
M_0.5_S_0.5_	50	50	1.0
M_0.6_S_0.4_	60	40	1.0
M_0.8_S_0.2_	80	20	1.0

Note: W/B ratio is the water to binder (MgO and SF) ratio by mass.

**Table 3:** Thermodynamic coefficients of the minerals (or species) in the MgO–SiO_2_–H_2_O system at 25 °C, 1 bar [30].

Minerals or Species	*S_m_*^θ^ (kJ·mol^−1^)	∆_f_*H_m_*^θ^ (kJ·mol^−1^)	∆_f_*G_m_*^θ^ (kJ·mol^−1^)
Mg^2+^ (aq)	−138.00	−466.85	−454.89
OH^−^ (aq)	−10.71	−230.02	−157.34
H^+^ (aq)	0.00	0.00	0.00
H_2_O (aq)	69.95	−285.83	−237.19
MgO (s)	26.95	−601.50	−569.23
SiO_2_ (Amorphous)	47.40	−903.20	−850.59
H_3_SiO_4_^−^ (aq)	112.55	−1426.16	−1253.98
H_2_SiO_4_^2−^ (aq)	−12.97	−1396.62	−1187.02
Mg(OH)_2_ (s)	63.14	−924.54	−833.56
M_3_S_2_H_2_ (Chrysotile)	221.30	−4361.66	−4034.02
M_3_S_4_H (Talc)	260.80	−5915.90	−5536.27

**Table 4:** Thermodynamic calculations for potential reactions.

ID	Minerals or Species	Reaction Equation	∆_r_*S*^θ^ (kJ·mol^−1^)	∆_r_*H*^θ^ (kJ·mol^−1^)	∆_r_*G*^θ^ (kJ·mol^−1^)	log*K*
①	MgO	MgO + 2H^+^ → Mg^2+^ + H_2_O	−95.00	−151.18	−122.85	21.52
②	H_2_SiO_4_^2−^	SiO_2_ + 2OH^−^ → H_2_SiO_4_^2−^	−38.95	−33.38	−21.75	3.81
③	H_3_SiO_4_^−^	SiO_2_ + OH^−^ + H_2_O → H_3_SiO_4_^−^	5.91	−7.11	−8.86	1.55
④	Mg(OH)_2_	Mg^2+^ + 2OH^−^ ↔ Mg(OH)_2_	222.56	2.35	−63.99	11.21
⑤	Chrysotile	3Mg^2+^ + 6OH^−^ + 2SiO_2_ ↔ 3MgO·2SiO_2_·2H_2_O + H_2_O	674.71	−60.42	−261.32	45.78
⑥	Talc	3Mg^2+^ + 6OH^−^ + 4SiO_2_ ↔ 3MgO·4SiO_2_·H_2_O + 2H_2_O	689.36	−94.09	−299.58	52.48

**Table 5:** Fitted parameters.

Sample ID	*K* (10^−6^ s^−1^)	*R* ^2^
M_0.2_S_0.8_	2.748	0.973
M_0.4_S_0.6_	2.040	0.988
M_0.5_S_0.5_	1.542	0.984
M_0.6_S_0.4_	1.577	0.971
M_0.8_S_0.2_	3.018	0.977

Note: *R*^2^ is the correlation coefficient.
